# Viability Test in Prediction of Response to Cardiac Resynchronization Therapy

**DOI:** 10.3390/jcm14155341

**Published:** 2025-07-29

**Authors:** Isidora Grozdic Milojevic, Nikola N. Radovanovic, Jelena Petrovic, Dragana Sobic-Saranovic, Vera Artiko

**Affiliations:** 1 Center for Nuclear Medicine and PET, University Clinical Center of Serbia, 11000 Belgrade, Serbia; jelena_petrovic18@yahoo.com (J.P.); dragana.sobic-saranovic@med.bg.ac.rs (D.S.-S.); vera.artiko@med.bg.ac.rs (V.A.); 2 Faculty of Medicine, University Belgrade, 11000 Belgrade, Serbia; 3 Pacemaker Center, University Clinical Center of Serbia, 11000 Belgrade, Serbia

**Keywords:** SPECT MPI, CRT response, prediction

## Abstract

**Background/Objectives**: This study aimed to evaluate myocardial scar burden and distribution, as well as other nuclear imaging parameters, in predicting cardiac resynchronization therapy (CRT) responses and long-term outcomes in patients selected for CRT with ischemic HF etiology. **Methods**: Seventy-one patients were prospectively included. They all had NYHA class II/III despite optimal medical therapy, LVEF ≤ 35%, wide QRS complexes, and ischemic HF etiology. All were indicated for de novo CRT implantation and underwent a SPECT MPI viability test prior to CRT implantation. Two-dimensional echocardiography was performed one day before CRT implantation and 6 months after the intervention. The follow-up examination was conducted six months after the CRT implantation and, after 5 years, patients underwent a telephone follow-up to assess survival. **Results**: Most patients (85%) were male, with an average age of 66.26 ± 9.25 yrs. SPECT MPI revealed large myocardial scars (44.53 ± 20.94%) with high summed rest scores (SRSs) of 25.02 ± 11.29 and low EFs of 26.67 ± 7.71%. At the 6-month follow-up, after the CRT implantation, the NYHA class significantly changed and 35% of the patients were classified as CRT responders. The only difference between responders and non-responders was in the SRS and myocardial scar size (*p* < 0.001). A scar size of 19.5% was an optimal cutoff for the prediction of CRT response (AUC 0.853, Sn 85% and 1-sp 94%). **Conclusions**: SPECT MPI parameters are valuable in predicting responses and long-term survival in patients with CRT. Patients with myocardial scars of less than 19.5% may be suited to CRT and experience better cardiovascular survival.

## 1. Introduction

The prevalence of diagnosed heart failure (HF) is about 1–2% of adults [1,2,3]. The lifetime risk of developing HF is 20% for individuals of 40+ years of age and is increasing, with coronary artery disease being the most common cause [1,2,3,4,5,6,7]. After the initial diagnosis, the course of the disease is unfavorable, accompanied by frequent hospitalizations, impaired quality of life, and a high mortality rate [8,9]. Cardiac resynchronization therapy (CRT) is a treatment option for patients with symptomatic heart failure with reducedejection fraction (HFrEF) despite optimal medical therapy and those who have wide QRS complexes in their ECG [10,11]. However, up to 40% of patients do not respond to CRT [12]. The reasons are multifactorial, and predicting who will respond remains a challenge [2]. The literature results show that patients with larger myocardial scars have markedly reduced clinical responses. Thus, the evaluation of myocardial viability prior to CRT is crucial.

Myocardial viability is a condition where dysfunctional heart muscle, resulting from acute or chronic ischemia, still has the capacity to regain normal systolic function following revascularization. Damage caused by ischemia can lead to irreversible myocardial injury; however, the heart also undergoes adaptive responses, allowing the affected myocardium to remain in a dormant state within an ischemic environment, with the potential to recover once blood flow is restored [1,2,3,4,5,6,7].

Single-photon emission tomography (SPECT) myocardial perfusion imaging is informative, available, and economical for the evaluation of myocardial viability and scar size (scar burden). It uses [^99m^Tc]-Tc-MIBI, a lipophilic compound with considerable cardiac affinity, which is distributed in the myocardium proportionally to the regional coronary flow. It enters the myocyte via passive diffusion through the cell and mitochondrial membrane of a viable and hibernating cardiomyocyte. The higher sensitivity of SPECT is likely due to the reliance of the tracer’s uptake on myocyte membrane integrity, which may be preserved even after the loss of myocyte contractile reserve.

SPECT imaging is simple to perform and provides numerous potentially predictive variables, such as quantitative scores and a precise volumetric evaluation of left ventricular function (end-diastolic volume (EDV), end-systolic volume (ESV), and left ventricular ejection fraction (LVEF)). One quantitative score is the summed rest score (SRS), a variable used to measure cardiac perfusion at rest in cardiac SPECT studies.

This study aimed to evaluate the value of myocardial scar burden and distribution, as well as other nuclear imaging parameters, in predicting CRT responses and long-term outcomes in patients selected for CRT with ischemic HF etiology.

## 2. Materials and Methods

Seventy-one patients with an indication for de novo CRT device implantation (with or without defibrillator function—CRT-P or CRT-D) were prospectively included in this study. They were all symptomatic (NYHA class II or III) despite optimal medical therapy, with reduced left ventricular ejection fraction (LVEF ≤ 35%), wide QRS complexes (LBBB QRS morphology and QRS duration > 130 ms or non-LBBB QRS morphology and QRS duration ≥ 150 ms), and ischemic HF etiology. All patients underwent coronary angiography. Etiology was considered ischemic in the presence of significant coronary artery disease (≥50% stenosis in 1 or more of the major epicardial coronary arteries), a history of myocardial infarction with electrocardiographic evidence, prior percutaneous coronary intervention, or prior coronary artery bypass graft surgery. Patients with a recent myocardial infarction (<3 months) or non-ambulatory NYHA class IV were excluded.

This prospective study included a routine follow-up examination of patients who came for a clinical visit six months after pacemaker implantation to assess clinical and echocardiographic improvement. Additionally, a long-term follow-up was conducted five years after pacemaker implantation. This follow-up method proved valuable for patients residing far from the clinic and those who did not attend regular check-ups. During this telephone consultation, all patients were contacted to evaluate the occurrence of any major adverse cardiovascular events (MACEs). MACEs were defined to include five specific events: cardiovascular death, myocardial infarction, stroke, repeat revascularization procedures, and hospitalization due to heart failure. The information regarding MACEs was subsequently verified by reviewing the patients’ medical records.

This work was performed according to the ethical principles of the Helsinki Declaration, and informed consent was obtained from all participants included in the follow-up. This study was approved by the Ethics Committee of the Faculty of Medicine, Center of Nuclear Medicine, University of Belgrade (IRB 668/6; 19 April 2018).

### 2.1. Nuclear Medicine Imaging, Data Reconstruction, and Image Analysis

One month before CRT implantation, all patients underwent single-photon emission tomography myocardial perfusion imaging (SPECT MPI) with [^99m^Tc]-Tc-MIBI to identify viable myocardium. Firstly, nitroglycerine (0.4 mg) was sublingually administered to all the patients. Heart rate and blood pressure were measured at baseline and 10 min following nitroglycerine administration. Then, 555 MBq (15 mCi) of [^99m^Tc]-Tc-MIBI was injected. The SPECT data were acquired during the rest, 45–60 min after the i.v. injection of [^99m^Tc]-Tc-MIBI.

All data were collected in the supine position using a single-head SPECT camera (e.cam; Siemens, Munich, Germany) with the following parameters: a high resolution, low energy, parallel-hole collimator, 180 degrees, and anterior noncircular orbit (45 degrees right anterior oblique to 45 degrees left posterior oblique). Images were taken in 64 projections in step-and-shoot mode, with 15s per projection, zoom at 1.45, and a matrix size of 64 × 64 × 16. The data were gated for 8 frames per cardiac cycle, with a T 50% R-R window and an energy window set at 140T 10% KeV. The quality of the data was assessed visually using the Siemens E Soft v10 Processing Workstation (version: Syngo MI P > 10.100.1510.1604 > VB10B).

Transaxial tomograms were generated from the gated projection data, reconstructed with the filtered back-projected algorithm, and reoriented to obtain oblique angle tomograms parallel to the long and short axes of the left ventricle. The reconstructed data were projected as myocardial tomographic slices in the short axis, vertical long axis, and horizontal long axis views. The data were analyzed with 4D-MSPECT. Following the guidelines of the American Society of Nuclear Cardiology, the American College of Cardiology, and the American Heart Association, the myocardium was divided into 17 segments [13]. A 5-point scoring system was used to assess the MIBI uptake (4 = no uptake/background only, 3 = severe reduction in uptake, 2 = moderate reduction in uptake, 1 = mild reduction in uptake, and 0 = normal uptake). The regional wall motion was assessed visually, while the functional parameters were derived from 4D-MSPECT: end-diastolic volume (EDV), end-systolic volume (ESV), and ejection fraction (EF) summed rest score (SRS).

The scar extent was calculated by dividing the total number of segments (17) by the number of scar segments; the result is expressed as %. The findings were interpreted separately by two nuclear medicine physicians who are highly experienced in nuclear cardiology. Consensus was reached in cases of discrepancy.

### 2.2. Echocardiography

Transthoracic 2-dimensional (2D) echocardiography was performed in all patients one day before CRT implantation and 6 months after the intervention. LV volumes and LVEF were calculated from conventional apical 2- and 4-chamber images using the biplane Simpson technique.

### 2.3. CRT Implantation

All procedures were performed under local anesthesia at the cardiac catheterization laboratory of the Pacemaker Center of the University Clinical Center of Serbia. For venous access, the cephalic vein cut-down technique (whenever possible) and subclavian/axillary vein puncture were used. The right atrial and ventricular leads were positioned conventionally. The preferred position for the left ventricular lead was a lateral or posterolateral tributary of the coronary sinus. The type of device (CRT-P or CRT-D) was chosen according to the preoperative characteristics of the patient, primarily according to age, HF etiology, and the presence of ventricular arrhythmias. Device programming was not mandated by a specific protocol but at the discretion of the implanting physician. Before discharge from the hospital, the electrocardiographic optimization of atrioventricular and interventricular delays was always performed.

Clinical evaluation included an assessment of NYHA functional class and exercise capacity and was performed before and six months after CRT implantation in all patients. After a six-month follow-up, the patients were divided into two groups, CRT responders and non-responders, assessed according to changes in certain clinical and echocardiographic parameters. A CRT responder was a patient whose LVEF increased by 5% or more and/or whose ESV decreased by at least 15% and who improved in at least one class category in the NYHA functional classification at follow-up. In this study, the CRT responder had to meet the stated echocardiographic and clinical criteria [5].

### 2.4. Statistical Analysis

The results are expressed as mean ± SD. Data comparison was performed using the paired and unpaired Student’s *t*-test for continuous variables. Categorical variables are expressed as numbers and percentages, and differences across the groups were assessed using the X^2^ test. Since this was a single-center study with a small number of participants (71), we took great care to ensure that the data were complete, so we werenot missing any data. Uni- and multivariable logistic regression analyses were performed to determine the relationship between potential risk factors at baseline and no response to CRT. All variables entered the multivariable stage, irrespective of the results of the univariable analyses. Thus, the multivariable analysis included the following: demographic factors (age and gender), clinical variables, and imaging variables (EF, EDV, ESV, scar size, scar localization, and SRS). We reported adjusted odds ratios (ORs) with their corresponding 95% confidence intervals (CIs).

The optimal scar extent needed to predict response to CRT was determined using receiver operating characteristic (ROC) curve analysis. For the ROC curve analysis, we used the SPSS 29 statistical program, which has functions for generating ROC curves and computing optimal cutoffs based on Youden’s index.

Survival estimates were calculated using the Kaplan–Meier method. A log-rank test was used to compare survival between groups. Cox proportional hazard models were used to assess multiple predictors of survival. A 2-sided *p*-value of less than 0.05 was considered significant.

## 3. Results

The study cohort comprised 71 patients (mean age 66.26 ± 9.25 years), predominantly male. The NYHA class was II or III (see Table 1).

**Figure 1:**
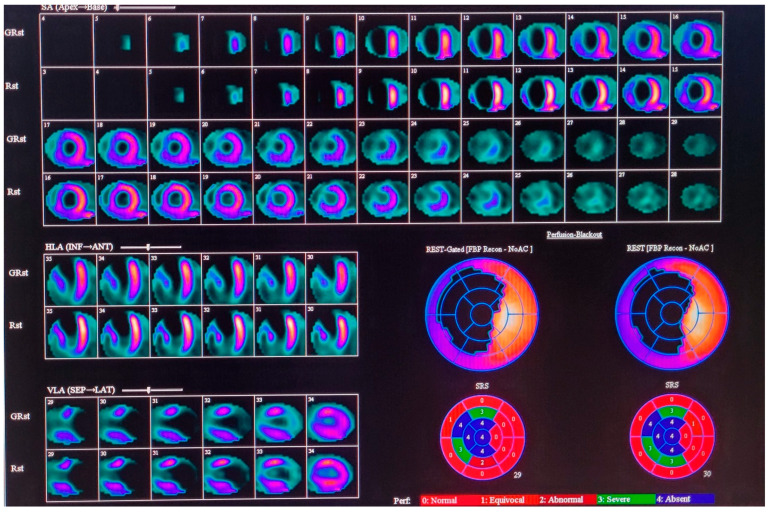
SPECT MPI of a patient revealing non-viable apex, apical, and medial segments of the anterior wall and an apical segment of the septal wall.

Imaging (SPECT MPI) revealed non-viable myocardium mainly in the inferior wall, septum, anterior wall, and apex (see Figure 1). The scar burden was large (44.53 ± 20.94%), with mean EFs of around 26.67 ± 7.71% (Table 2). Echocardiography and nuclear assessments of EFs were not significantly different (*p* > 0.05).

Left ventricular dilation was common, with increased EDV and ESV, along with a high SRS (25.02 ± 11.29). After the diagnostic procedures mentioned, CRT-D was implanted in 36 patients and CRT-P in 35. All procedures were successful, with LV lead placement in the targeted lateral or posterolateral tributary of the coronary sinus. Perioperative complications occurred in four patients (5.6%), including one pneumothorax and three lead dislodgements, which were managed successfully.

**Scheme 1:**
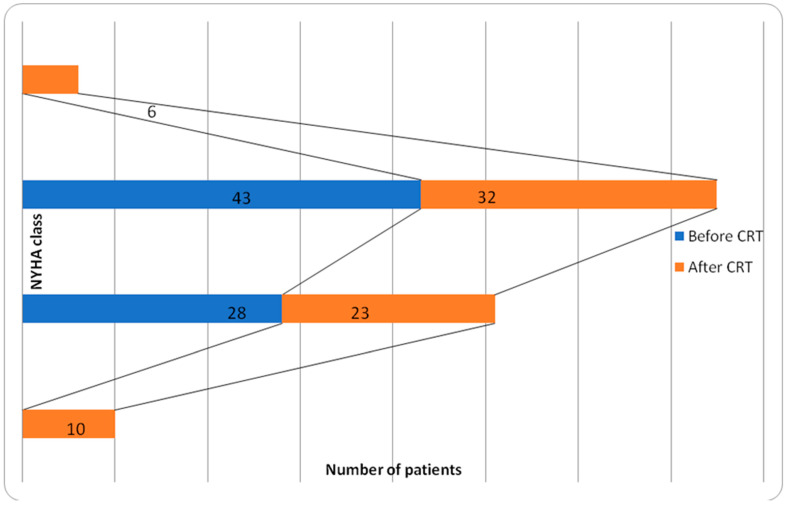
NYHA class distribution before and after CRT.

At six months, EF increased from 25.52 ± 7.74% to 29.40 ± 11.39% (*p* = 0.005) and the NYHA class changed significantly (*p* = 0.011, X^2^test) (Scheme 1). Twenty-five patients were classified as CRT responders (35%).

No significant differences in age, gender, medication, comorbidities, EF, EDV, or ESV were observed between responders and non-responders. However, SRSs and scar size were notably higher in non-responders (30.78 ± 10.13 vs. 17.57 ± 6.35, *p* < 0.001, CI 8.23–18.19; 55.03 ± 16.53 vs. 32.31 ± 14.73, *p* < 0.001) (Table 3 and Table 4).

Linear regression showed that scar size inversely correlated with EF at follow-up (*p* = 0.001). Scar localization differed significantly: responders commonly had scars in the apex (*p* < 0.001, X^2^ test) and inferior wall (*p* < 0.001, X^2^ test). Responders usually had implanted CRT-D, while non-responders usually had implanted CRT-P (*p* < 0.001).

Both scar size and SRSs predicted CRT response in the univariate analysis (OR 0.919 and 0.953, respectively; *p* = 0.008 and *p* = 0.003). Anterior and septal scar localization also predicted responses (Table 5). A multivariate analysis did not identify independent predictors, probably because of collinearity or overfitting.

**Figure 2:**
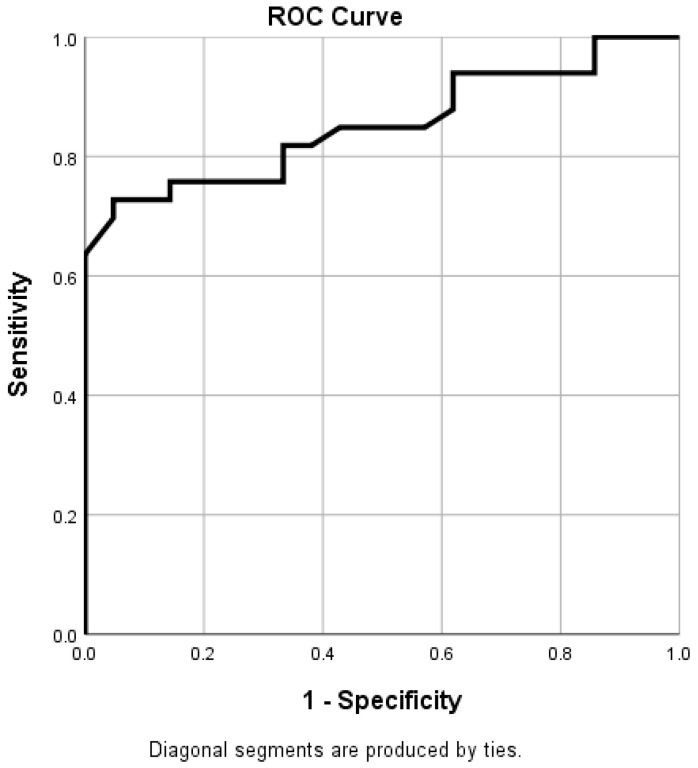
ROC curve for optimal scar size % in predicting CRT response.

Over five years, 37% of the patients remained stable, 63% had MACEs (49% experienced cardiovascular death, 2% MI, 2% stroke, 7% hospitalization due to HF worsening, and 3% hospitalization for CABG/PCI). The ROC analysis determined a scar size cutoff of 19.5% for predicting CRT responses, with an AUC of 0.853, sensitivity of 85%, and 1-sp of 94% (Figure 2).

**Figure 3:**
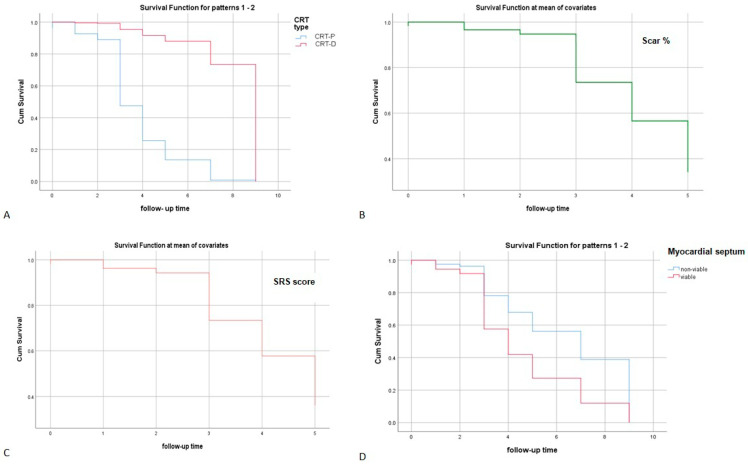
Cox regression analysis indicated better survival outcomes in patients with: (**A**). Implanted CRT-D (exp (B) 15.6, *p* = 0.008, CI 2.076–117.231), (**B**). Smaller scar burden (exp (B) 0.953, *p* = 0.003, CI 0.924–0.984), (**C**). Lower SRS score (exp (B) 0.919, *p* = 0.008, CI 0.856–0.978), (**D**). Less frequent scar localization in myocardial septum (exp (B) 0.325, *p* = 0.048, CI 0.106–0.002).

A survival analysis indicated that the patients with scar burdens below 19.5% had better cardiovascular survival (*p* = 0.039, log-rank). The patients with CRT-D also had improved survival outcomes (exp (B) 15.6, *p* = 0.008, CI 2.076–117.231). Responders at six months had better survival if they had a smaller scar burden (exp (B) 0.953, *p* = 0.003, CI 0.924–0.984), lower SRSs (exp (B) 0.919, *p* = 0.008, CI 0.856–0.978), and less frequent septal scar localization (exp (B) 0.325, *p* = 0.048, CI 0.106–0.002) (Figure 3).

## 4. Discussion

Among CRT recipients, ischemic cardiomyopathy is associated with poor outcomes, which can be attributed to the global scar burden, multiple scar segments, and regional ischemia that may affect the remodeling response to biventricular pacing. Furthermore, the left ventricular pacing site and its location relative to scar and or ischemia may result in ineffectual resynchronization and suboptimal clinical responses [14,15,16,17,18,19,20,21,22,23]. Thus, numerous procedures are used for the evaluation of myocardial viability.

This assessment was first performed using 2D echocardiography. However, low repeatability and suboptimal acoustic windows in approximately 20% of the patients have led to this procedure being considered suboptimal in evaluating CRT [24,25].

Magnetic resonance evaluation of LV function also has many shortcomings. It is expensive, difficult to access, time-consuming, and inappropriate for use in patients who have metal implants, who are claustrophobic, or who have poor renal function [26,27].

On the other hand, SPECT MPI is an accessible, economical, and informative method. However, there are still concerns regarding its use, mainly due to the use of ionizing radiation [28].

This prospective study included 71 consecutive patients with ischemic heart failure (NYHA class II or III), depressed LVEF (<35%), and substantial electrical LV dyssynchrony. According to the SPECT MPI, non-viable myocardium was usually present in more than two localizations. This information allowed the cardiologists to position the lead outside the scar zone.

Thepatients came to a follow-up examination 6 months after the pacemaker implantation. NYHA class significantly changed (*p* = 0.011), and EF improved (25.52 ± 7.74, vs. 29.40 ± 11.39, *p* = 0.005), so twenty-five patients were classified as CRT responders. Baseline demographic and clinically relevant parameters did not differ significantly between CRT responders and non-responders. Responders had significantly less scar burden and lower SRSs. We also wanted to define the optimal cutoff scar value to predict CRT response. Based on the ROC curve analysis, this cutoff was 19.5% scarring (area under the curve of 0.853, sensitivity of 85%, and 1-sp of 94%).

The patients with less scarring than this cutoff had better survival according to the Kaplan–Meier analysis (log-rank test, 4.28; *p* = 0.039). Better survival was present in responders who had lower SRSs, less scar burden, and less frequent septal scar localization.

At the five-year follow-up, 37% of the patients remained stable, and 63% had MACEs.

Ypenburg and Adelstein [6,23] noted that responders were more frequently patients with non-ischemic HF etiology. Significant improvement at 6 months after CRT was observed in those with ischemic HF, with 53% of the patients classified as responders. In our study, the response rate was lower (35%). This may be because we included only patients with ischemic HFrEF etiology and predominantly advanced symptoms of the disease. Furthermore, in our population, every fourth patient had atrial fibrillation. On the other hand, by definition, the responder had to achieve both clinical and echocardiographic improvement, which is not always the case in the literature. This precise definition of CRT response can be useful as it facilitates a comprehensive approach—integrating symptomatic subjective evaluation (NYHA class) with structural and functional cardiac metrics (quantitative assessment using echocardiography)—to objectively predict and assess treatment outcomes, thereby enhancing patient management.

Many studies have reported that patients with non-lateral scar localization have better responses to CRT, as most CRT LV leads are positioned in the LV lateral wall, where scars are infrequently present in radionuclide MPI imaging. Hence, the scar location by itself does not significantly impact the CRT outcome, but mismatches between scar location and LV lead position do [2,17,21,29,30,31,32].

Other studies have shown that CRT can recover LV function through reverse LV remodeling if, preoperatively, there is a reduced contractile function in the interventricular septum, i.e., asymmetry in workload between the septum and lateral wall and preserved septal viability [30,31,32]. In our study, smaller SRSs (*p* = 0.008), smaller scars (*p* = 0.003), and less frequent septal scar localization were more frequent in CRT responders. Aldestein et al. reported similar results in their study, where the extensive scar burden met significant statistical criteria and affected clinical, functional, and survival outcomes after CRT [23,29].

Ypenburg et al. found that 35% scarring can predict CRT responses [6]. Adelstein et al. expressed their results as the number of non-viable segments or SRSs; therefore, we converted our SRS number into percentages to enable a comparison of the data. If we assume that the maximum SRS can be 68 (if each of the 17 segments of the left ventricle myocardium receives a score of 4), then a patient with an SRS of 27 can have a maximum of six non-viable segments, meaning the scar % in that study is around 39.7%. Given the above, the criteria in our study are somewhat stricter, considering that the cutoff was 19.5%. This indicates that, in the population of patients with ischemic cardiomyopathy, SPECT viability assessment should be performed as early as possible, when the percentage of scar tissue is still low, thereby increasing the likelihood of a good response and improved survival.

This study included 71 patients; conversely, Ypenburg et al. included 61 patients, and Aldestein et al. included 67 patients. Additionally, their studies used different radiopharmaceuticals. Aldestein et al. used SPECT MPI with [^201^Tl], while Ypenburg et al. used [^18^F]FDG. Furthermore, Ypenburg et al. defined a responder as someone who experienced an improvement in at least one NYHA class, whereas Adelstein et al. considered responders to be those with an increase in ejection fraction (EF) greater than 15% after CRT implantation [6,23]. For this reason, these results are not entirely comparable.

Regardless of the scar percentage, the initial degree of cardiac function impairment is important for long-term outcomes, as it reflects both the level of autonomic imbalance and the degree of deterioration in control and compensatory mechanisms. Scar burden remains a risk factor for sudden cardiac death even with improved LVEF, especially in patients with implanted CRT-P. It seems that many other variables affect CRT response, and the functional integrity of the sympathetic nerve terminals in this area may play an important role [33].

Five years after CRT implantation, 37% of the patients were stable. Unfortunately, 49% died, while 14% had another type of MACE (myocardial infarction, stroke, repeat revascularization procedure, or hospitalization due to worsening of heart failure).

In our study population, responders with CRT-D had better survival. CRT-P devices are primarily used throughout our country, mainly driven by economic considerations. The primary indication for CRT-D implantation is when it is essential for the secondary prevention of sudden cardiac death, particularly in younger patients with ischemic heart disease and especially if revascularization is incomplete. Additionally, CRT-D implantation is preferred in patients with better functional status, as they are at a higher risk of sudden cardiac death than those with advanced heart failure, who are more likely to succumb to progressive heart failure or non-cardiac causes. Therefore, we can explain these results.

Similar results were cited by Barra et al. [34]. After a mean follow-up period of 41.4 ± 29.0 months, patients with ischemic cardiomyopathy had better survival when receiving CRT with a defibrillator than those who received CRT without a defibrillator (HR: 0.76; CI 0.62–0.92; *p* = 0.005). Layeva et al. also had concordant results.CRT-D was associated with lower total mortality and the composite endpoints of total mortality or heart failure hospitalization, as well as total mortality or hospitalization for MACEs (HR 0.71) (all *p*< 0.001) [35]. However, there were no differences in outcomes between CRT-D and CRT-P in patients with non-ischemic cardiomyopathy [34,35].

SPECT MPI is a practical, automated technique used to identify LV dyssynchrony, LVEF, LV volumes, ischemia, viability, and scar tissue [36,37,38,39,40,41]. Multiple studies have indicated that the average effective dose associated with the [^99m^Tc]-Tc-MIBI viability test is approximately 6–8 mSv, comparable to the dose from coronary angiography, which averages around 7.15 ± 3.4 mSv. In contrast, [^18^F]FDG viability PET scans deliver a significantly lower radiation dose of about 4.9 mSv, although their availability is limited in some hospitals [42,43,44].

Recent studies concerning quantitative radiomics analysis have provided new insights into survival prediction [45,46]. In a study by Feeny etal. [47], a machine learning model was developed for CRT outcome prediction by enrolling 925 patients with nine clinical features (QRS morphology, QRS duration, New York Heart Association classification, left ventricular ejection fraction and end-diastolic diameter, sex, ischemic cardiomyopathy, atrial fibrillation, and epicardial left ventricular lead). The machine learning model outperformed the conventional guideline with an increased AUC of 0.70 vs. 0.65 (*p*-value < 0.02) and an increased event-free survival with a concordance index of 0.61 vs. 0.56 (*p*-value < 0.001). In a study by Sabouri et al., machine learning models were developed to predict left ventricular contractile patterns, leading to a success rate of 76% compared with traditional patient selection in predicting treatment outcome based on contractile patterns [48]. However, this new, promising, growing area of science is not yet ready to enter the clinical phase and replace the presence of a doctor.

This study has some limitations. Firstly, we employed a small sample size. Based on the sample size calculation for significance levels (α = 0.05; power = 80%), a sample size should be around 100 patients. The post hoc power analysis was 79%, which is generally considered acceptable and close to the commonly recommended threshold of 80%. Increasing the number of participants in future studies can provide even greater confidence. Additionally, we need to address potential overfitting in the multivariable models, as some variables were significant in the univariate analysis but not in the multivariable analysis. Secondly, we lacked PET/CT imaging, which has higher sensitivity in evaluations of myocardial viability. Thirdly, this study utilized data from a single center. Future research should be conducted in multicenter studies to enable broader conclusions and to assess the generalizability of the findings.

## 5. Conclusions

Nuclear medicine procedures are accessible, quick, and straightforward to perform, providing valuable insights into the assessment of myocardial viability. These techniques involve a manageable level of radiation exposure and provide valuable predictive parameters that are useful for the selection of patients who will benefit from CRT. A scar burden of less than 19.5% can predict good CRT responses and good cardiovascular survival in patients with an ischemic etiology of heart failure. In addition, SRS, CRT-D, and preserved myocardial septum viability were found to positively influence cardiovascular survival. Thus, nuclear medicine imaging should be performed before CRT implementation to improve patient selection.

## Figures and Tables

**Table 1:** Patient characteristics.

Variable	Overall Value
Age	66.26 ± 9.25
Male gender	61 (85%)
NYHA class	II 40% (28 pts) III 60% (43 pts)
QRS duration (ms)	164 ± 21
True LBBB	50 (70%)
EF% (ultrasound measurement)	24.93 ± 7.84
Medication *	
Diuretics	93%
ACE inhibitors	82%
Beta-blockers	53%

* Majority of patients had two or more medications.

**Table 2:** SPECT MPI characteristics.

Variable	Overall Value
LVEF %	26.67 ± 7.71
LVEDV	277.34 ± 93.17
LVESV	208.21 ± 83.48
SRS	25.02 ± 11.29
Scar %	44.53 ± 20.94

**Table 3:** Differences in characteristics between CRT responders and non-responders.

Variable	CRT Responders (%)	CRT Non-Responders (%)	*p*
Age	66.96 ± 9.21	66.76 ± 8.45	0.900
Male gender	84%	87%	0.786
Atrial fibrillation	22%	24%	0.810
Medication *			
Diuretics	92%	93%	0.970
ACE inhibitors	87%	89%	0.830
Beta-blockers	58%	56%	0.860

* Majority of patients had two or more medications.

**Table 4:** Differences in imaging variables between CRT responders and non-responders.

Variable	Responder	Non-Responder	*p*
SPECT parameters			
LVEF%	26.20 ± 8.76	27.21 ± 7.49	0.690
LVEDV	265.81 ± 113.43	281.22 ± 82.49	0.651
LVESV	200.75 ± 102.71	210.11 ± 73.44	0.760
SRS	17.57 ± 6.34	30.78 ± 10.13	**0.001**
Scar %	32.31 ± 14.73	55.03 ± 16.53	**0.001**
EF % (ultrasound measurement)	25.08 ± 8.81	25.81 ± 7.05	0.600
Periprocedural complications	3 (12%)	1 (2%)	0.294

**Table 5:** Univariate and multivariate logistic regression.

		Univariate	Analysis		Multivariate	Analysis
Variable	OR	95% CI	*p*	OR	95% CI	*p*
Gender	0.661	0.186–2.345	0.521	0.000	0.000–1.100	0.884
Age	0.984	0.926–1.046	0.602	1.575	0.640–3.850	0.320
EF	1.009	0.946–1.077	0.780	0.410	0.080–1.930	0.260
EDV	0.997	0.988–1.006	0.509	1.400	0.840–2.330	0.197
ESV	0.996	0.985–1.007	0.456	0.654	0.314–1.240	0.195
SRS	0.919	0.865–0.978	**0.008**	0.917	0.205–4.640	0.889
Anterior scar localization	0.059	0.006–0.552	**0.013**	0.977	0.000–1.600	0.976
Septal scar localization	0.315	0.106–0.992	**0.048**	0.001	0.130–4.000	0.225
Inferior scar localization	0.436	0.052–3.630	0.440	1106.9	0.000–8.000	0.897
Lateral scar localization	0.167	0.022–1.250	0.080	82,039	0.000–8.100	0.893
Inferolateral scar localization	0.000	0.00–2.130	0.986	0.000	0.000–8.520	0.953
Apical scar localization	0.11	0.023–0.537	**0.020**	0.000	0.100–6.010	0.780
Scar %	0.953	0.924–0.984	**0.003**	0.917	0.271–7.900	0.889

## Data Availability

No new data were created or analyzed in this study. Data sharing is not applicable to this article.

## Abbreviations

The following abbreviations are used in this manuscript:

HF	Heart failure
LVEF	Left ventricular ejection fraction
HFrEF	Heart failure with reduced ejection fraction
CRT	Cardiac resynchronization therapy
SPECT	Single-photon emission tomography
MPI	Myocardial perfusion imaging
MACE	Major adverse cardiovascular event
EDV	End-diastolic volume
ESV	End-systolic volume
SRS	Summed rest score
OR	Odds ratio
ROC curve	Receiver operating characteristic curve
